# Perceived Interpersonal Racism and Incident Stroke Among US Black Women

**DOI:** 10.1001/jamanetworkopen.2023.43203

**Published:** 2023-11-10

**Authors:** Shanshan Sheehy, Hugo J. Aparicio, Julie R. Palmer, Yvette Cozier, Vasileios-Arsenios Lioutas, Julie G. Shulman, Lynn Rosenberg

**Affiliations:** 1 Slone Epidemiology Center, Boston University, Boston, Massachusetts; 2 Boston University Chobanian & Avedisian School of Medicine, Boston, Massachusetts; 3 Boston Medical Center, Boston, Massachusetts; 4 Boston University Center for Antiracist Research, Boston, Massachusetts; 5 Boston University School of Public Health, Boston, Massachusetts; 6 Beth Israel Deaconess Medical Center, Boston, Massachusetts

## Abstract

**Importance:**

Black individuals in the US experience stroke and stroke-related mortality at younger ages and more frequently than other racial groups. Studies examining the prospective association of interpersonal racism with stroke are lacking.

**Objective:**

To examine the association of perceived interpersonal racism with incident stroke among US Black women.

**Design, Setting, and Participants:**

The Black Women’s Health Study, a prospective cohort study of 59 000 Black women from across the US, assessed the longitudinal association between perceived interpersonal racism and stroke incidence. Stroke-free participants were followed up from 1997 until onset of stroke, death, loss to follow-up, or the end of the study period (December 31, 2019). Cox models were used to estimate hazard ratios (HRs) and 95% CIs, adjusting for major confounders, including education, neighborhood socioeconomic environment, and cardiometabolic factors. Data analysis was performed from March 2021 until December 2022.

**Exposure:**

On a questionnaire completed in 1997, participants reported experiences of racism in everyday life and when dealing with situations that involved employment, housing, and interactions with police.

**Main Outcomes and Measures:**

Strokes were identified through self-report on biennial questionnaires, medical records adjudication, and linkage with the National Death Index.

**Results:**

In 1997, 48 375 Black women (mean [SD] age, 41 [10] years) provided information on perceived interpersonal racism and were free of cardiovascular disease and cancer. During the 22 years of follow-up, 1664 incident stroke cases were identified; among them, 550 were definite cases confirmed by neurologist review and/or National Death Index linkage. Multivariable HRs for reported experiences of racism in all 3 domains of employment, housing, and interactions with police vs no such experiences were 1.38 (95% CI, 1.14-1.67), a 38% increase, for all incident cases and 1.37 (95% CI, 1.00-1.88) for definite cases. For comparisons of women in the highest quartile of everyday interpersonal racism score vs women in the lowest quartile, multivariable HRs were 1.14 (95% CI, 0.97-1.35) for analyses that included all incident stroke and 1.09 (95% CI, 0.83-1.45) for analyses that included definite cases only.

**Conclusions and Relevance:**

In this study, Black women who reported experiences of interpersonal racism in situations involving employment, housing, and interactions with police appeared to have an increased risk of stroke, even after accounting for demographic and vascular risk factors, suggesting that the high burden of racism experienced by Black US women may contribute to racial disparities in stroke incidence.

## Key Points

**Question:**

Is perceived interpersonal racism associated with stroke risk?

**Findings:**

In this cohort study of 48 375 US Black women, those who reported experiencing interpersonal racism in situations involving employment, housing, and the police had an estimated 38% increased risk of stroke compared with women who reported no such experiences.

**Meaning:**

These findings suggest that the high burden of racism experienced by Black US women may contribute to racial disparities in stroke incidence.

## Teaser

This cohort study examines the association of perceived interpersonal racism with incident stroke among US Black women.

## Introduction

Black US individuals are especially vulnerable to stroke, with a 2- to 3-fold higher stroke incidence^1,2,3,4^ and 1.2-fold higher stroke mortality than White US individuals.^5^ Black women, in particular, experience stroke and stroke-related mortality at higher rates and earlier onset than women in any other racial group.^6,7^

Racism in the US (both interpersonal racism and structural racism) disproportionally affects Black individuals. Racism is a complex construct and exists in multiple forms and at multiple levels.^8,9,10,11^ Direct evidence linking racism with incident stroke is quite limited. The Reasons for Geographic and Regional Differences in Stroke study^12^ provided important evidence that risk of stroke increased with increasing numbers of adverse social determinants of health, but racism was not directly assessed. The Multi-Ethnic Study of Atherosclerosis^13^ reported no association of race-based discrimination with incident cardiovascular disease (CVD), but the association specifically with stroke was not reported. Some studies^14,15,16^ have reported subclinical CVD associated with discrimination, although the results were inconsistent. The Black Women’s Health Study (BWHS), begun in 1995, asked a series of validated questions on experiences of racism in 1997, allowing for a prospective examination of the association of perceived interpersonal racism with incident stroke among Black women for 22 years of follow-up.

## Methods

The Boston University Medical Campus institutional review board approved this cohort study. Study participants provided written informed consent. This report follows the Strengthening the Reporting of Observational Studies in Epidemiology (STROBE) reporting guidelines for observational studies.

### Study Population

The BWHS^17^ is an ongoing, prospective cohort study that initially enrolled 59 000 participants through *Essence Magazine* and Black professional organizations (94% of the BWHS participants came from the *Essence Magazine* subscription lists). The BWHS is the largest contemporary cohort of Black women in the US, and most Black women were from 17 states across the mainland US, including California, Colorado, Georgia, Illinois, Indiana, Kansas, Louisiana, Maryland, Massachusetts, Michigan, Minnesota, New Jersey, New York, Oklahoma, South Carolina, Virginia, and Wisconsin, and Washington, District of Columbia. BWHS participants live in neighborhoods with a wide range of neighborhood socioeconomic status (SES). Black women aged 21 to 69 years (median age at baseline, 38 years) enrolled in 1995 by completing health questionnaires. In the initial questionnaire, respondents provided data on demographic characteristics, socioeconomic factors, medical conditions, and lifestyle factors. All but 5% of BWHS participants were born in the US.^17^ Every 2 years, participants update health information on follow-up mailed and web health questionnaires. Follow-up through 2019 is complete for 86.2% of person-years.

### Incident Stroke

Stroke cases were ascertained through (1) biennial BWHS questionnaires, which asked participants to report whether a physician had given them a diagnosis of stroke and the year of the diagnosis; (2) stroke medical records, which were reviewed and by a committee of neurologists (H.J.A., V.-A.L., and J.G.S.); and (3) the National Death Index (NDI).^18^ On the 1995 BWHS questionnaire, participants were asked, “Has a doctor ever told you that you have any of the following conditions” (yes or no), and among the list was stroke. If yes, participants were asked to mark the age at which stroke was first diagnosed (<30, 30-39, 40-49, or ≥50 years). On the 1997 BWHS questionnaire and onward, participants were specifically asked, “If a doctor has told you that you had a stroke, please indicate when it was first diagnosed.” Self-reported stroke by the participant was followed by collection of detailed diagnostic information from medical records, including from inpatient hospitalizations and outpatient clinic visits. Among 1976 BWHS participants who reported a physician-diagnosed stroke, we were able to obtain releases and relevant portions of the medical record for 618. All medical records, including neuroimaging where available, were independently reviewed by an adjudication committee of vascular neurologists (H.J.A., V.-A.L., and J.G.S.), using the World Health Organization definition of stroke^19^: a new focal (or at times global) neurological impairment of sudden onset and of presumed vascular origin.^19^ These included ischemic stroke, intracerebral hemorrhage, and subarachnoid hemorrhage. In addition, retinal and spinal cord infarction were included in this definition. Events not meeting this definition but with clinical evidence consistent with stroke, including (1) stroke that had been aborted by thrombolysis or mechanical thrombectomy and (2) symptoms lasting less than 24 hours with visible infarction on neuroimaging (ie, transient ischemic attack with demonstration of an ischemic lesion on magnetic resonance diffusion-weighted imaging), were also classified as stroke events. A reviewer with uncertainty about the status of the event forwarded it to a second reviewer. A joint discussion then took place between 2 to 3 reviewers to achieve a consensus (H.J.A., V.-A.L., or J.G.S.). Uncertainty was resolved through committee meetings for adjudication.

Appropriate medical records were obtained for 618 of 1976 participants who reported stroke. Among them, 450 (73%) were confirmed through adjudication and 168 were disconfirmed. Interrater reliability between the independent reviewers for adjudication of a confirmed stroke event was excellent (κ = 92%). Fatal stroke was ascertained through linkage with the NDI (*International Statistical Classification of Diseases and Related Health Problems, Tenth Revision *[*ICD-10*] codes I60-I69).

For the present analysis, the primary end point includes stroke confirmed by medical records review or NDI linkage, or self-reported stroke cases for which medical records could not be obtained. Stroke confirmed by medical records review or NDI were considered as definite cases. The secondary end point was restricted to definite cases only.

### Perceived Interpersonal Racism

BWHS questions on racism and/or discrimination were adapted from an instrument developed by Williams et al,^20^ designed to measure perceived interpersonal racism. We assessed perceived interpersonal racism experienced by the individual in everyday life and perceived interpersonal racism when dealing with situations that involved employers, housing, and police (eTable 1 in Supplement 1).^21,22,23^ The present analysis is based on 1997 responses to the perceived interpersonal racism questions,^20,24^ which represent perceived racism at midlife for most of the participants.

Five questions addressed interpersonal racism in everyday activities, such as, “People act as if they think you are dishonest,” with 5 responses ranging from never (scored as 0) to almost every day (scored as 4). The score for interpersonal racism in everyday life was the mean of responses to all 5 questions (possible scores, 0-4) and was categorized into quartiles. Three other questions asked whether the respondent had ever experienced discrimination owing to her race in employment, housing, and the police.^21,22,23,24,25^ The score for perceived racism in institutional settings was the sum of positive responses to the 3 questions about employment, housing, and police (possible scores, 0, 1, 2, and 3). The BWHS has previously reported associations of perceived interpersonal racism with increased risks of type 2 diabetes^26^ and obesity,^22^ decreased subjective cognitive function,^23^ and other outcomes.^21,24,27,28,29,30,31,32,33,34^

### Covariate Assessment

Covariates were chosen a priori on the basis of factors known to be associated with increased risk of stroke. We included age (continuous), body mass index (calculated as weight in kilograms divided by height in meters squared; ≤24.9, 25-29.9, 30-34.9, and ≥35), education level (≤12, 13-15, 16, and ≥17 years), neighborhood socioeconomic status (SES) in quintiles,^35^ vigorous physical activity (<1, 1-4 hours, or ≥5 hours per week), cigarette smoking (never, current <15 cigarettes per day, current ≥15 cigarettes per day, quit <10 years ago, and quit ≥10 years ago), history of diabetes (yes or no), history of hypertension (yes or no), history of coronary heart disease (yes or no), history of hyperlipidemia (yes or no), history of depression (yes or no), insurance status (have health insurance, yes or no), and indicators for health care utilization (had visited a doctor or nurse practitioner for a general physical and/or had your own regular physician or nurse practitioner, yes or no). All covariate values were taken from the 1997 questionnaire, the point at which the exposure, perceived interpersonal racism, was measured.

Hypertension, reported by participants on biennial questionnaires, was defined as physician-diagnosed hypertension together with use of an antihypertensive medication or diuretic, or use of an antihypertensive alone. In a prior validation study,^36^ 138 of 139 self-reports of hypertension (99%) were confirmed by medical records. Women who reported a diagnosis of diabetes occurring at age 30 years or older were considered to have type 2 diabetes. In a validation study,^37^ 217 of 229 women (94%) who reported diabetes were confirmed by their physicians to have type 2 diabetes.

### Statistical Analysis

Multivariable adjusted Cox proportional hazard models were used to estimate hazard ratios (HRs) and 95% CIs. For each study participant, follow-up was from 1997 until the onset of incident stroke, death, loss to follow-up, or the end of study follow-up (December 31, 2019), whichever came first. On the basis of known risk factors for stroke, we decided a priori to examine potential effect modification by baseline covariates, including age, residence in the so-called Stroke Belt (ie, Alabama, Arkansas, Georgia, Louisiana, Mississippi, North Carolina, South Carolina, and Tennessee),^38^ educational level, and neighborhood SES. We used the Wald test to assess the significance of interaction. We also derived Kaplan-Meier survival curves for each of racism categories in the primary analysis. All analyses were performed using SAS statistical software version 9.4 (SAS Institute). All analyses involved 2-sided tests with a significance level of *P* < .05. Data analysis was performed from March 2021 until December 2022.

## Results

In 1997, 48 375 women (mean [SD] age, 41 [10] years) provided information on perceived interpersonal racism and were free of CVD (843 participants) and cancer (1615 participants). Compared with women in the lowest quartile of perceived interpersonal racism score, those in the highest quartile were similar with respect to neighborhood SES, body mass index, smoking, and physical activity (Table 1). They were more likely to have a higher educational level. A total of 27 155 women (59%) perceived racism in employment, 16 109 (35%) perceived racism in housing, and 11 046 (24%) perceived racism in interactions with police. In the present study, 5159 participants (11%) reported yes to all 3 questions on perceived racism in employment, housing, and interactions with police, and 13 951 women (30%) reported no to all 3 questions. In this study, the correlation coefficient between everyday racism and housing, employment, and police scores was 0.36. Women who reported racism in employment, housing, and the police were more likely to live in high SES neighborhoods, less likely to live in the South, and more likely to have a higher educational level than those who reported no such experience (Table 1).

**Table 1.** Age-Standardized Baseline Characteristics in Black Women’s Health Study Participants by Quartiles of Perceived Interpersonal Racism

Variable	Participants, No. (%)^a^			
Perceived interpersonal racism in everyday life	Quartile 1 (n = 8960)	Quartile 2 (n = 13 676)	Quartile 3 (n = 13 769)	Quartile 4 (n = 11 970)
Age, mean (SD), y	40.6 (11)	40.6 (11)	40.5 (11)	40.6 (11)
Neighborhood socioeconomic status				
Lowest quintile	2527 (28)	3460 (25)	3552 (26)	3352 (28)
Highest quintile	1612 (18)	2585 (19)	2561 (19)	2226 (19)
Geographic region				
Northeast	2509 (28)	3665 (27)	3566 (26)	3124 (26)
South	2867 (32)	4445 (33)	4296 (31)	3615 (30)
Midwest	1882 (21)	3050 (22)	3236 (24)	2873 (24)
West	1702 (19)	2462 (18)	2616 (19)	2310 (19)
Body mass index, mean (SD)^b^	27.4 (6)	27.6 (6)	28.0 (7)	28.7 (7)
Smoking				
Never smoker	6021 (67)	9067 (66)	8895 (65)	7385 (62)
Past smoker, quit <10 y ago	27 (0.3)	41 (0.3)	41 (0.3)	36 (0.3)
Past smoker, quit ≥10 y ago	1532 (17)	2571 (19)	2754 (20)	2526 (21)
Current smoker, <15 cigarettes/d	1066 (12)	1559 (11)	1570 (11)	1472 (12)
Current smoker, ≥15 cigarettes/d	314 (4)	438 (3)	496 (4)	539 (5)
Vigorous physical activity, No. of h/wk				
<1	1344 (15)	2174 (16)	2217 (16)	1951 (16)
1-4	3047 (34)	4923 (36)	5150 (37)	4166 (35)
≥5	1165 (13)	1819 (13)	1831 (13)	1652 (14)
Alcohol, current	2419 (27)	3788 (28)	4021 (29)	3759 (31)
Hypertension	1523 (17)	2448 (18)	2534 (18)	2418 (20)
Diabetes	448 (5)	684 (5)	647 (5)	646 (5)
Education, y				
≤12	2061 (23)	2421 (18)	2258 (16)	2155 (18)
≥17	1613 (18)	2968 (22)	3305 (24)	2729 (23)
Perceived interpersonal racism in employment, housing and by the police	No to all (n = 13 951)	Yes to 1 (n = 15 478)	Yes to 2 (n = 11 437)	Yes to all 3 (n = 5159)
Age, mean (SD), y	40.3 (10)	40.3 (10)	40.3 (10)	40.3 (10)
Neighborhood socioeconomic status				
Lowest quintile	3948 (28)	4055 (26)	2814 (25)	1336 (26)
Highest quintile	2274 (16)	2817 (18)	2310 (20)	1047 (20)
Geographic region				
Northeast	3627 (26)	3993 (26)	3134 (27)	1419 (28)
South	4771 (34)	5232 (34)	3317 (29)	1290 (25)
Midwest	3111 (22)	3483 (23)	2653 (23)	1264 (25)
West	2400 (17)	2740 (18)	2322 (20)	1187 (23)
Body mass index, mean (SD)^b^	27.9 (7)	27.9 (7)	27.9 (7)	28.1 (7)
Smoking				
Never smoker	9417 (68)	10 154 (66)	7308 (64)	3157 (61)
Past smoker, quit <10 y ago	42 (0.3)	46 (0.3)	34 (0.3)	15 (0.3)
Past smoker, quit ≥10 y ago	2400 (17)	2863 (19)	2345 (21)	1130 (22)
Current smoker, <15 cigarettes/d	1590 (11)	1857 (12)	1304 (11)	655 (13)
Current smoker, ≥15 cigarettes/d	488 (4)	573 (4)	446 (4)	201 (4)
Vigorous physical activity, No. of h/wk				
<1	2051 (15)	2523 (16)	1910 (17)	872 (17)
1-4	4785 (34)	5557 (36)	4323 (38)	1945 (38)
≥5	1744 (13)	2105 (14)	1601 (14)	753 (15)
Alcohol, current	3725 (27)	4411 (29)	3500 (31)	1656 (32)
Hypertension	2539 (18)	2802 (18)	2024 (18)	918 (18)
Diabetes	698 (5)	789 (5)	515 (5)	232 (5)
Education, y				
≤12	3278 (24)	2616 (17)	1590 (14)	727 (14)
≥17	2623 (19)	3343 (22)	2779 (24)	1377 (27)

^a^ Values are standardized to the age distribution of the study.

^b^ Body mass index is calculated as weight in kilograms divided by height in meters squared.

During follow-up from 1997 through 2019, a total of 1664 incident strokes were identified. As shown in Table 2 and eFigures 1 and 2 in Supplement 1, the age-adjusted HR for stroke was 1.28 (95% CI, 1.09-1.50; *P* for trend < .001) for women in the highest quartile of perceived interpersonal racism in everyday life vs those in the lowest quartile. The multivariable HR was 1.14 (95% CI, 0.97-1.35; *P* for trend = .03). When comparing women who experienced racism in employment, housing, and the police with those with no such experience, the age-adjusted HR for stroke was 1.42 (95% CI, 1.18-1.70; *P* for trend < .001). The multivariable HR was 1.38 (95% CI, 1.14-1.67; *P* for trend < .001), or a 38% increase, for women who answered yes to all 3 domains (employment, housing, and police) vs women who answered no to all 3 domains.

**Table 2.** Multivariable Associations of Perceived Interpersonal Racism With Incident Stroke

Variable	No. of incidents/No. of person-years	Incidence rate/1000 person-years	HR (95% CI)	
			Model 1^a^	Model 2^b^
Perceived interpersonal racism in everyday life (n = 1664)^c^				
Quartile 1	341/166 745	2.05	1 [Reference]	1 [Reference]
Quartile 2	445/262 587	1.69	0.90 (0.77-1.05)	0.90 (0.77-1.06)
Quartile 3	434/267 158	1.62	0.99 (0.84-1.16)	0.98 (0.84-1.15)
Quartile 4	444/230 944	1.92	1.28 (1.09-1.50)	1.14 (0.97-1.35)
*P* value for trend	NA	NA	<.001	.03
Perceived interpersonal racism in employment, housing, and the police (n = 1530)^d^				
No to all	401/266 551	1.50	1 [Reference]	1 [Reference]
Yes to 1	464/298 094	1.56	1.11 (0.96-1.29)	1.12 (0.97-1.30)
Yes to 2	433/220 721	1.96	1.23 (1.06-1.44)	1.27 (1.09-1.48)
Yes to 3	232/99 211	2.34	1.42 (1.18-1.70)	1.38 (1.14-1.67)
*P* value for trend	NA	NA	<.001	<.001

Abbreviations: HR, hazard ratio; NA, not applicable.

^a^ Model 1 was stratified by age (continuous).

^b^ Model 2 was stratified by age (continuous), neighborhood socioeconomic status (quintiles), participant’s education level (≤12, 13-15, 16, or ≥17 years), body mass index (calculated as weight in kilograms divided by height in meters squared; ≤24.9, 25-29.9, 30-34.9, and ≥35), vigorous physical activity (<1, 1-4, and ≥5 hours per week), smoking (never, current <15 cigarettes per day, current ≥15 cigarettes per day, former quit <10 years ago, and former quit ≥10 years ago), diabetes (yes or no), hypertension (yes or no), family history of myocardial infarction (yes or no), hyperlipidemia (yes or no), depression (yes or no), insurance status (yes or no), and health care utilization (yes or no).

^c^ *P* = .11 for proportional hazard assumption for perceived interpersonal racism in everyday life.

^d^ *P* = .83 for proportional hazard assumption for perceived interpersonal racism in employment, housing, and by the police.

We repeated the analyses restricted to 550 definite cases that were confirmed through neurologist adjudication and/or the NDI (Table 3). For perceived interpersonal racism in everyday life, there was no association with risk of stroke (multivariable adjusted HR, 1.09; 95% CI, 0.83-1.45; *P* for trend = .66). For women who reported interpersonal racism in all 3 of the domains of employment, housing, and police compared with none of the domains, the multivariable adjusted HR was 1.37 (95% CI, 1.00-1.88; *P* for trend = .05) (Table 3). In a sensitivity analysis that restricted definite strokes to only those with medical records adjudicated by study neurologists, the comparable HR was 1.61 (95% CI, 1.09-2.37) (eTable 2 in Supplement 1).

**Table 3.** Multivariable Associations of Perceived Interpersonal Racism With Incident Definite Stroke

Variable	No. of incidents/No. of person-years	Incidence rate/1000 person-years	HR (95% CI)	
			Model 1^a^	Model 2^b^
Perceived interpersonal racism in everyday life (n = 550)^c^				
Quartile 1	124/166 646	0.74	1 [Reference]	1 [Reference]
Quartile 2	164/262 286	0.63	1.04 (0.81-1.33)	1.04 (0.81-1.34)
Quartile 3	141/266 910	0.53	1.01 (0.77-1.31)	0.99 (0.76-1.29)
Quartile 4	121/230 781	0.52	1.20 (0.91-1.58)	1.09 (0.83-1.45)
*P* value for trend	NA	NA	.27	.66
Perceived interpersonal racism in employment, housing and by the police (n = 504)^d^				
No to all	134/266 412	0.50	1 [Reference]	1 [Reference]
Yes to 1	165/297 843	0.55	1.16 (0.91-1.48)	1.17 (0.91-1.49)
Yes to 2	135/220 483	0.61	1.16 (0.90-1.50)	1.21 (0.93-1.56)
Yes to 3	70/99 051	0.71	1.37 (1.00-1.87)	1.37 (1.00-1.88)
*P* value for trend	NA	NA	.06	.05

Abbreviations: HR, hazard ratio; NA, not applicable.

^a^ Model 1 was stratified by age (continuous).

^b^ Model 2 was stratified by age (continuous), neighborhood socioeconomic status (quintiles), participant’s education level (≤12, 13-15, 16, and ≥17 years), body mass index (calculated as weight in kilograms divided by height in meters squared; ≤24.9, 25-29.9, 30-34.9, ≥35), vigorous physical activity (<1, 1-4, and ≥5 hours per week), smoking (never, current <15 cigarettes per day, current ≥15 cigarettes per day, former quit <10 years ago, and former quit ≥10 years ago), diabetes (yes or no), hypertension (yes or no), family history of myocardial infarction (yes or no), hyperlipidemia (yes or no), depression (yes or no), insurance status (yes or no), and health care utilization (yes or no).

^c^ *P* = .01 for proportional hazard assumption for perceived interpersonal racism in everyday life.

^d^ *P* = .56 for proportional hazard assumption for perceived interpersonal racism in employment and housing and by the police.

Across strata of various subgroups, perceived interpersonal racism in employment, housing, and the police was consistently associated with elevated stroke risk (eTable 3 in Supplement 1). For perceived interpersonal racism in everyday life, the HR was most increased among participants in the lowest quartile of neighborhood SES (*P* for interaction = .001) (eTable 3 in Supplement 1).

## Discussion

In this large, prospective cohort study of 48 375 Black women, we found that participants who reported experiencing interpersonal racism in 3 institutional settings had an estimated 38% increased risk of stroke compared with women who reported no such experiences. The elevated stroke risk was present in analyses of all incident strokes, as well as in analyses restricting to definite strokes. For perceived interpersonal racism in everyday life, stroke risk was elevated in an analysis that included all stroke cases, but there was no evidence of an increased risk of stroke based on analyses of the smaller number of definite cases.

Disproportionate numbers of Black US individuals face multiple adverse experiences over the life course, including racism and poverty; these are recognized as social determinants of health.^39,40^ Racism may act as a psychosocial stressor and thereby elevate systemic inflammation, impair endothelial function, and dysregulate the hypothalamic-pituitary-adrenal axis.^41,42,43^ Previous studies have linked perceived interpersonal racism with worse mental health outcomes,^41^ higher risk of hypertension,^24,44,45,46,47,48^ increased systolic blood pressure,^48^ unhealthy behavior and lifestyles,^49,50^ higher allostatic load,^51,52,53^ higher inflammatory markers,^54^ hormone dysregulation,^55,56^ and shorter telomere length.^27,57^ Although several large prospective cohort studies have investigated stroke risk factors among Black US individuals, direct evidence about perceived racism and incident stroke is very limited. The Jackson Heart Study^14^ reported higher risk of subclinical CVD associated with discrimination, but did not examine stroke end points. The Multi-Ethnic Study of Atherosclerosis^13^reported no association of racial discrimination with risk of all CVD, but associations with stroke were not reported. Our study provides direct evidence on perceived racial discrimination at the interpersonal level in relation to subsequent occurrence of incident stroke.

### Strengths and Limitations

Strengths of our study include the prospective cohort design with a large sample of Black women, wide geographic distribution, long duration of follow-up from 1997 until 2019, large number of incident strokes, and detailed information on potential confounding factors. Our study also has some limitations. First, the concept of racism is complex, and racism exists in multiple forms and at multiple levels. Structural racism^58^ (ie, the ways societies reinforce racial differences in access to housing, education, employment, health care, and criminal justice) may also affect stroke risk and is not captured by questions about interpersonal racism. Second, our primary stroke end point included self-reported stroke. BWHS participants responded to the question, “Has a doctor ever told you that you have stroke?” Medical records were not available for all stroke events, either because the participant declined to sign a medical records release or because the appropriate hospital was not identified or did not have the records. A team of vascular neurologists performed a careful review of records that were obtained. The confirmation rate of 73% is similar to that reported in other studies of stroke.^59^ Prior studies have shown that the question, “Has a doctor ever told you that you have stroke?” has good sensitivity and specificity for identifying patients in the community with a diagnosis of stroke.^59,60^

Third, perceived racism captures an individual’s perceptions of an experience of racism and relies on a participant’s self-report. Perceived racism is inevitably subject to measurement error. An individual who experiences racism may overreport or underreport these sensitive events or change their interpretations and perceptions of these experiences over time. Because self-reported information on stroke was collected prospectively by biennial questionnaire, it is highly unlikely that stroke events were reported differentially with respect to the perceived interpersonal racism level reported in 1997. However, it is possible that BWHS participants who survived a stroke with severe deficits may have been less likely to respond.

Fourth, despite our efforts to control for confounders and risk factors for stroke, our study is observational and may still have been subject to unmeasured and residual confounding. For example, in our study, we were unable to adjust for history of atrial fibrillation, a risk factor for stroke. However, given the extensive list we included for major confounder adjustment, it is highly unlikely that our study will have such a strong unmeasured confounder.

Fifth, our study consisted of Black women with high educational levels compared with the general population of Black women. Participants in the BWHS needed to have sufficient literacy to complete health questionnaires every 2 years. BWHS participants represent the educational levels of Black US women, with underrepresentation of women with the lowest level of education (ie, <12 years). Future studies among Black women with lower levels of education are needed.

## Conclusions

In this investigation of perceived interpersonal racism in relation to incident stroke, experiences of interpersonal racism in everyday life were associated with higher risk of incident stroke, although there was no association for the smaller group of definite strokes. However, in both analyses, individuals who reported experiencing interpersonal racism in situations concerning housing, employment, and the police appeared to have an increased risk, estimated to be 38%, compared with women who reported no such experiences. It is possible that the high burden of racism experienced by Black US individuals may contribute to racial disparities in stroke incidence.

## Notes

Supplement 1. **eTable 1.** BWHS Data Collection on Racism **eTable 2.** Multivariable Associations of Perceived Interpersonal Racism With Incident Definite Stroke Confirmed by Medical Records **eTable 3.** Associations of Perceived Interpersonal Racism With Incident Stroke by Subgroups **eFigure 1.** Kaplan-Meier Survival Curve for the Association Between Perceived Racism in Everyday Life With Incident Stroke **eFigure 2.** Kaplan-Meier Survival Curve for the Association Between Perceived Racism in Job, Housing and by the Police With Incident Stroke

Supplement 2.
Data Sharing Statement
