# Supplementary material for: Perceived Interpersonal Racism and Incident Stroke Among US Black Women
Source: JAMA Netw Open. 2023 Nov 10;6(11):e2343203. doi: 10.1001/jamanetworkopen.2023.43203 (PMC10638652; doi:10.1001/jamanetworkopen.2023.43203)
Supplement: Supplement 1. — eTable 1. BWHS Data Collection on Racism eTable 2. Multivariable Associations of Perceived Interpersonal Racism With Incident Definite Stroke Confirmed by Medical Records eTable 3. Associations of Perceived Interpersonal Racism With Incident Stroke by Subgroups eFigure 1. Kaplan-Meier Survival Curve for the Association Between Perceived Racism in Everyday Life With Incident Stroke eFigure 2. Kaplan-Meier Survival Curve for the Association Between Perceived Racism in Job, Housing and by the Police With Incident Stroke [file jamanetwopen-e2343203-s001.pdf]

Supplemental Online Content

Sheehy S, Aparicio HJ, Palmer JR, et al. Perceived interpersonal racism and incident stroke among US Black women. *JAMA Netw Open*. 2023;6(11):e2343203. doi:10.1001/jamanetworkopen.2023.43203

eTable 1. BWHS Data Collection on Racism
eTable 2. Multivariable Associations of Perceived Interpersonal Racism With Incident Definite Stroke Confirmed by Medical Records
eTable 3. Associations of Perceived Interpersonal Racism With Incident Stroke by Subgroups
eFigure 1. Kaplan-Meier Survival Curve for the Association Between Perceived Racism in Everyday Life With Incident Stroke
eFigure 2. Kaplan-Meier Survival Curve for the Association Between Perceived Racism in Job, Housing and by the Police With Incident Stroke

This supplemental material has been provided by the authors to give readers additional information about their work.

© 2023 Sheehy S et al. *JAMA Network Open.*

eTable 1. BWHS Data Collection on Racism

	Questions	Responses
**Perceived interpersonal racism at everyday life**	In your day-to-day life, how often have any of the following things happened to you?	
	You receive poorer service than other people at restaurants or stores.	Never, a few times a year, once a month, once a week, almost every day
	People act as if they think you are not intelligent.	
	People act as if they are afraid of you.	
	People act as if they think you are dishonest.	
	People act as if they are better than you.	
**Perceived interpersonal racism at employment, housing and by the police**	Have you ever been treated unfairly due to your race in any of the following circumstances?	
	Job (hiring, promotion, firing)	"Yes", "No"
	Housing (renting, buying, mortgage)	
	Police (stopped, searched, threatened)	

© 2023 Sheehy S et al. *JAMA Network Open.*

eTable 2. Multivariable Associations of Perceived Interpersonal Racism With Incident Definite Stroke Confirmed by Medical Records

	N/PY	Incidence rate Per 1000 person years	Model 1 HR (95% CI)	Model 2 HR (95% CI)
**Perceived interpersonal racism in everyday life**				
**Q1**	79/168207	0.47	Ref	Ref
**Q2**	107/264389	0.40	0.90 (0.65, 1.23)	0.89 (0.65, 1.22)
**Q3**	109/269162	0.40	0.97 (0.71, 1.34)	0.95 (0.69, 1.31)
**Q4**	94/233011	0.40	1.12 (0.80, 1.57)	1.02 (0.73, 1.43)
**p trend**			0.39	0.78
**Perceived interpersonal racism in employment, housing and by the police**				
**0 to all**	82/268294	0.31	Ref	Ref
**Yes to 1**	127/300115	0.42	1.47 (1.08, 2.00)	1.47 (1.08, 2.01)
**Yes to 2**	103/222693	0.46	1.44 (1.04, 1.99)	1.45 (1.04, 2.04)
**Yes to 3**	55/100192	0.55	1.66 (1.13, 2.44)	1.61 (1.09, 2.37)
**p trend**			0.009	0.02

Model 1 stratified by age (continuous). Model 2 stratified by age (continuous), neighborhood socioeconomic status (quintiles), participant's education level (≤12, 13-15, 16, ≥17 years), body mass index (≤24.9, 25-29.9, 30-34.9, ≥35 kg/m^2), vigorous physical activity (<1hr, 1-4 hrs, ≥5 hrs per week), smoking (never, current

© 2023 Sheehy S et al. *JAMA Network Open.*

<15 cigs/day, current ≥15 cigs per day, former, quit <10 years ago, former, quite ≥10 years ago), diabetes (yes, no), hypertension (yes, no), family history of myocardial infarction (yes, no), hyperlipidemia (yes, no), depression (yes, no), insurance status (yes, no), and health care utilization (yes, no).

© 2023 Sheehy S et al. *JAMA Network Open.*

eTable 3. Associations of Perceived Interpersonal Racism With Incident Stroke by Subgroups

	Stroke Belt Yes	Stroke Belt No	Age <65 years	Age ≥65 years	Neighborhood SES Q1	Neighborhood SES Q5	Education <16 years	Education ≥16 years
	**Multivariable HR (95% CI)**	**Multivariable HR (95% CI)**	**Multivariable HR (95% CI)**	**Multivariable HR (95% CI)**	**Multivariable HR (95% CI)**	**Multivariable HR (95% CI)**	**Multivariable HR (95% CI)**	**Multivariable HR (95% CI)**
**Perceived interpersonal racism in everyday life**								
**Q1**	Ref	Ref	Ref	Ref	Ref	Ref	Ref	Ref
**Q2**	0.87 (0.64, 1.20)	0.92 (0.76, 1.10)	0.92 (0.76, 1.12)	0.85 (0.64, 1.13)	0.93 (0.67, 1.30)	0.74 (0.49, 1.10)	0.94 (0.77, 1.14)	0.82 (0.62, 1.07)
**Q3**	0.93 (0.67, 1.29)	1.02 (0.85, 1.23)	0.98 (0.81, 1.19)	1.02 (0.76, 1.38)	1.36 (0.99, 1.87)	0.92 (0.61, 1.39)	0.99 (0.81, 1.21)	0.94 (0.72, 1.24)
**Q4**	1.20 (0.86, 1.65)	1.15 (0.95, 1.39)	1.17 (0.97, 1.41)	0.98 (0.68, 1.41)	1.37 (0.98, 1.91)	0.95 (0.62, 1.46)	1.21 (0.99, 1.48)	1.00 (0.75, 1.33)
**P trend**	0.21	0.06	0.04	0.02	0.01	0.88	0.01	0.62
**P for interaction**		0.89		0.39		0.001		0.08

© 2023 Sheehy S et al. *JAMA Network Open.*

Perceived interpersonal racism in employment, housing and by the police								
	**Multivariable HR (95% CI)**	**Multivariable HR (95% CI)**	**Multivariable HR (95% CI)**	**Multivariable HR (95% CI)**	**Multivariable HR (95% CI)**	**Multivariable HR (95% CI)**	**Multivariable HR (95% CI)**	**Multivariable HR (95% CI)**
**No to all**	Ref	Ref	Ref	Ref	Ref	Ref	Ref	Ref
**Yes to 1**	1.32 (1.00, 1.76)	1.07 (0.89, 1.28)	1.10 (0.93, 1.30)	1.15 (0.83, 1.59)	1.01 (0.75, 1.36)	0.95 (0.61, 1.47)	1.12 (0.93, 1.34)	1.11 (0.85, 1.45)
**Yes to 2**	1.14 (0.83, 1.57)	1.33 (1.11, 1.59)	1.21 (1.01, 1.44)	1.48 (1.07, 2.03)	1.31 (0.96, 1.79)	1.39 (0.92, 2.08)	1.30 (1.07, 1.57)	1.23 (0.94, 1.62)
**Yes to 3**	1.14 (0.75, 1.74)	1.44 (1.16, 1.78)	1.30 (1.05, 1.61)	1.68 (1.13, 2.51)	1.28 (0.87, 1.90)	1.45 (0.89, 2.36)	1.32 (1.04, 1.68)	1.45 (1.06, 1.98)
**P trend**	0.50	<0.001	0.007	0.003	0.06	0.04	0.03	0.01
**P for interaction**		0.11		0.11		0.54		0.67

Stroke belt was defined as North Carolina, South Carolina, Georgia, Tennessee, Alabama, Mississippi, Arkansas, and Louisiana.

Model 1 stratified by age (continuous).

Model 2 stratified by age (continuous), neighborhood socioeconomic status (quintiles), participant's education level (≤12, 13-15, 16, >17 years), body mass index (≤24.9, 25-29.9, 30-34.9, ≥35 kg/m^2), vigorous physical activity (<1hr, 1-4 hrs, >5 hrs per week), smoking (never, current <15 cigs/day, current >15 cigs per day, former, quit <10 years ago, former, quite >10 years ago), diabetes (yes, no), hypertension (yes, no), family history of myocardial infarction (yes, no), hyperlipidemia (yes, no), depression (yes, no), insurance status (yes, no), and health care utilization (yes, no).

© 2023 Sheehy S et al. *JAMA Network Open.*

eFigure 1. Kaplan-Meier Survival Curve for the Association Between Perceived Racism in Everyday Life With Incident Stroke

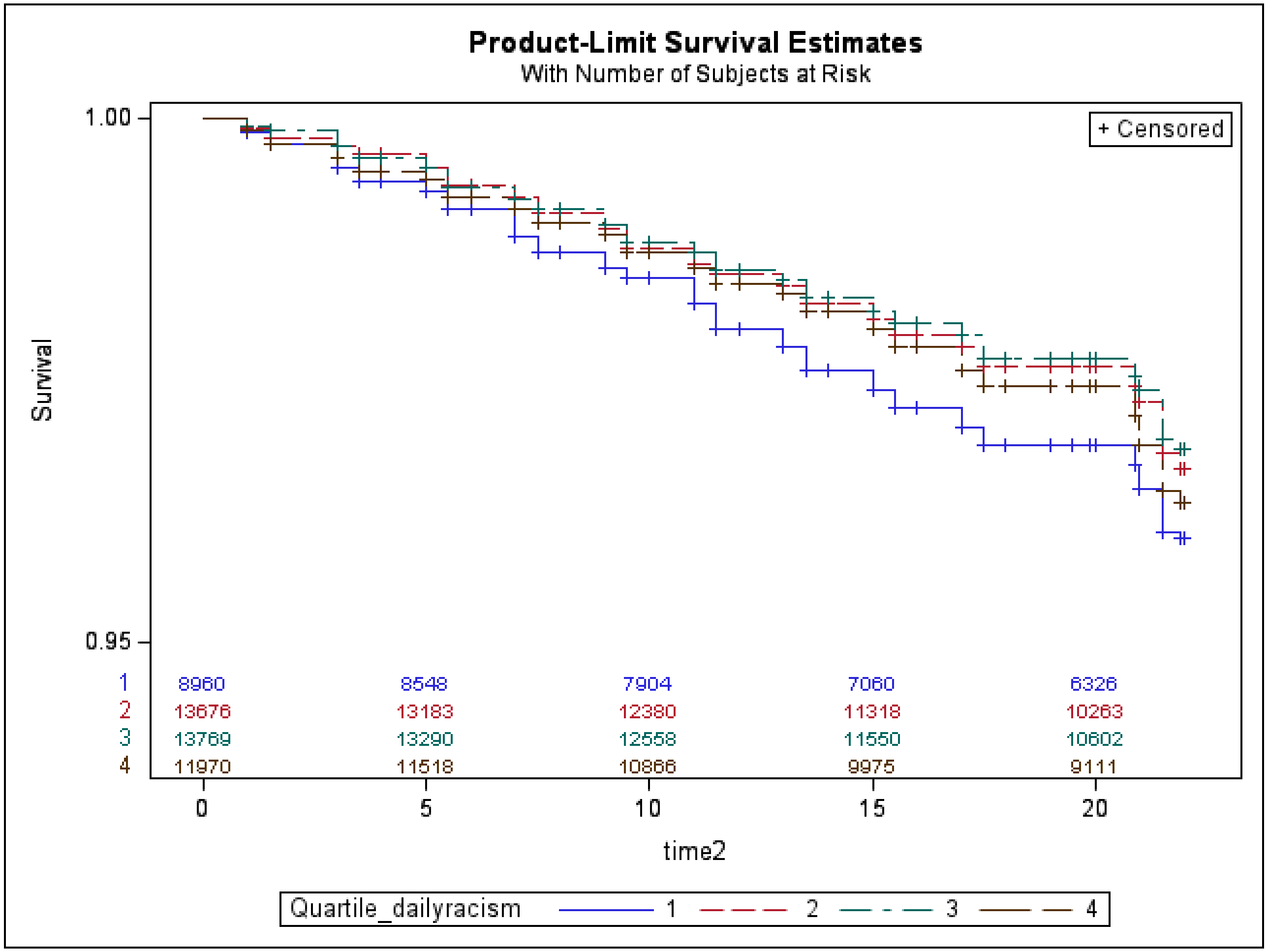

© 2023 Sheehy S et al. *JAMA Network Open.*

eFigure 2. Kaplan-Meier Survival Curve for the Association Between Perceived Racism in Job, Housing and by the Police With Incident Stroke

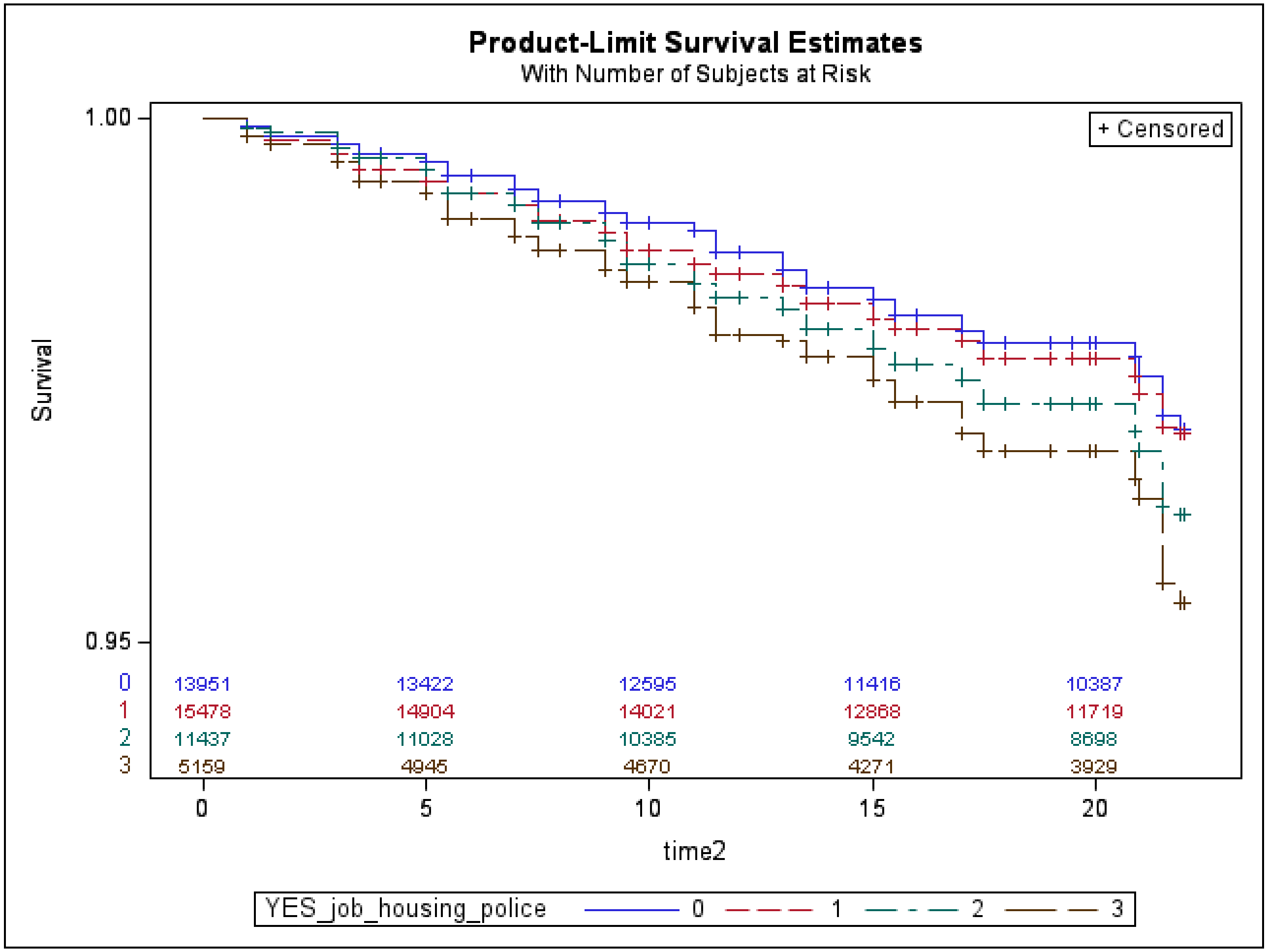

© 2023 Sheehy S et al. *JAMA Network Open.*
