# Supplementary material for: Perceived Interpersonal Racism and Incident Stroke Among US Black Women
Source: JAMA Netw Open. 2023 Nov 10;6(11):e2343203. doi: 10.1001/jamanetworkopen.2023.43203 (PMC10638652; doi:10.1001/jamanetworkopen.2023.43203)
Supplement: Supplement 2. — Data Sharing Statement [file jamanetwopen-e2343203-s002.pdf]

Data Sharing Statement

Sheehy. Perceived Interpersonal Racism and Incident Stroke Among US Black Women. *JAMA Netw Open*. Published November 14, 2023. doi:10.1001/jamanetworkopen.2023.43203

Data
Data available: No

Additional Information
Explanation for why data not available: Information on the procedure to obtain and access data from the Black Women's Health Study is described at http://www.bu.edu/bwhs under the information for Researchers.
